# Proposing Urothelial and Muscle In Vitro Cell Models as a Novel Approach for Assessment of Long-Term Toxicity of Nanoparticles

**DOI:** 10.3390/ijms21207545

**Published:** 2020-10-13

**Authors:** Matej Skočaj, Maruša Bizjak, Klemen Strojan, Jasna Lojk, Mateja Erdani Kreft, Katarina Miš, Sergej Pirkmajer, Vladimir Boštjan Bregar, Peter Veranič, Mojca Pavlin

**Affiliations:** 1 Group for nano and biotechnological applications, Faculty of Electrical Engineering, University of Ljubljana, SI-1000 Ljubljana, Slovenia; matej.skocaj@bf.uni-lj.si (M.S.); marusa.bizjak@mf.uni-lj.si (M.B.); klemen.strojan@gmail.com (K.S.); jasna.lojk@ffa.uni-lj.si (J.L.); vladimir.bregar@gmail.com (V.B.B.); 2 Institute of Pathophysiology, Faculty of Medicine, University of Ljubljana, SI-1000 Ljubljana, Slovenia; katarina.mis@mf.uni-lj.si (K.M.); sergej.pirkmajer@mf.uni-lj.si (S.P.); 3 Institute of Cell Biology, Faculty of Medicine, University of Ljubljana, SI-1000 Ljubljana, Slovenia; mateja.erdani@mf.uni-lj.si; 4 Institute of Biophysics, Faculty of Medicine, University of Ljubljana, SI-1000 Ljubljana, Slovenia

**Keywords:** nanotoxicology, in vitro L6 rat skeletal muscle cell line, urothelium in vitro, differentiation, cell signaling, ROS

## Abstract

Many studies evaluated the short-term in vitro toxicity of nanoparticles (NPs); however, long-term effects are still not adequately understood. Here, we investigated the potential toxic effects of biomedical (polyacrylic acid and polyethylenimine coated magnetic NPs) and two industrial (SiO_2_ and TiO_2_) NPs following different short-term and long-term exposure protocols on two physiologically different in vitro models that are able to differentiate: L6 rat skeletal muscle cell line and biomimetic normal porcine urothelial (NPU) cells. We show that L6 cells are more sensitive to NP exposure then NPU cells. Transmission electron microscopy revealed an uptake of NPs into L6 cells but not NPU cells. In L6 cells, we obtained a dose-dependent reduction in cell viability and increased reactive oxygen species (ROS) formation after 24 h. Following continuous exposure, more stable TiO_2_ and polyacrylic acid (PAA) NPs increased levels of nuclear factor Nrf2 mRNA, suggesting an oxidative damage-associated response. Furthermore, internalized magnetic PAA and TiO_2_ NPs hindered the differentiation of L6 cells. We propose the use of L6 skeletal muscle cells and NPU cells as a novel approach for assessment of the potential long-term toxicity of relevant NPs that are found in the blood and/or can be secreted into the urine.

## 1. Introduction

In the last three decades, various new nanomaterials and nanoparticles (NPs) have been implemented into diverse industrial and medical applications, exploiting the advantages of their small size, high reactivity, and other specific properties. Consequently, we are regularly exposed to different engineered NPs that are used as pigments (TiO_2_), are involved in food processing (food grade TiO_2_, SiO_2_), or are used as ingredients of cosmetics (TiO_2_, ZnO), with estimates that an average person in the USA is exposed to 1 mg/kg body weight of TiO_2_ per day [1]. In parallel, there are already several U.S. Food and Drug Administration (FDA)-approved biomedical NP formulations that are used for drug delivery or as contrast agents for MRI (e.g., magnetic NPs) [2]. Importantly, every new type of NP has to be appropriately characterized in terms of its physicochemical characteristics in the relevant physiological media [3,4]. NPs can trigger adverse reactions in the cells, and thus, it is crucial to assess potential short-term as well as long-term toxicity for both industrially relevant and biomedical NPs. Since most industrial NPs are not biodegradable and can persist and accumulate in tissues for longer time periods, some of the toxic effects of NPs might be evident only after long-time exposure, which implies the necessity for long-time studies and the need for appropriate cell culture models.

The mechanisms of NPs toxicity are very complex and diverse. NPs can induce lower proliferation rates, oxidative stress (ROS; reactive oxygen species formation), changes in cell metabolism and cell signaling, DNA damage, autophagy and lysosomal dysfunctions, and changes in the morphology, and they can also disrupt the cell cytoskeleton and hinder cell differentiation [5,6,7], depending on their properties, concentration, and exposure time. NPs can also trigger pro-inflammatory reactions through several mechanisms, including nuclear factor κB (NF-κB) activation, inflammasome activation, or others [8,9]. The toxicity of NPs can also be a result of ion leakage or a consequence of released toxic degradation products [10]. In addition, some studies used NPs concentrations that are much higher than physiologically relevant, which adds to sometimes confusing results on cytotoxicity.

Some of these toxicity mechanisms manifest only after prolonged exposure, which is often neglected during short-term in vitro studies but crucial for better understanding of prolonged environmental or medical exposure, which can result in an organ accumulation of NPs [11,12,13,14,15]. However, due to the variety of protocols, lack of standardized physicochemical characterization of NPs [3,4], and only a limited number of long-term studies [16,17,18,19,20,21,22,23,24,25,26,27,28,29,30,31,32,33,34,35,36,37,38], there is no general agreement about the potential long-term toxicity of several types of specific NPs. Long-term TiO_2_ NPs exposure has been shown to alter proliferation [38] as well as induce stress [38] and inflammatory responses [19,30,38]. On the other hand, the majority of the long-term studies using SiO_2_ NPs show that they are nontoxic, although some studies found that certain types of SiO_2_ NPs exhibit size- and charge-dependent cytotoxic effects [32,33] or can induce inflammation [29,32]. Moreover, several studies have indicated that cells have the ability to adapt to persistent stress to some extent [19,23]; however, there is little information on the possible chronic toxicity of NPs formulations at concentrations that do not induce acute toxicity.

Due to the lack of the appropriate models of differentiating and/or differentiated cells that would more accurately represent the in vivo condition, only a limited number of studies evaluated the long-term cytotoxic effects of NPs [16,17,18,19,20,21,22,23,24,25,26,27,28,29,30,31,32,33,34,35,36,37,38]. Thus, the great majority of long-term studies is performed on proliferative cells, which require repeated subcultivation [16,17,18,20,23,26,27,28,29,32,34,35,36,38] or are grown in bioreactors [31,32]. While the drawback of growing the cells in bioreactors is high cost, the repeated subcultivation leads to the redistribution of internalized NPs into daughter cells, which means less NPs and less toxic effects per cell. In in vivo conditions, the majority of cells are not dividing; therefore, the number of internalized NPs is not decreasing due to cell divisions, but stays the same or even increases, as one would expect in case of chronic exposure. Therefore, in vitro models of differentiating cells and highly differentiated cells more closely resemble properties of terminally differentiated tissue cells and could aid nanotoxicity testing, reduce experiments on animals, and enable a faster translation of new nanomaterials into practice.

In this study, we evaluate two well-characterized in vitro cell models for a long-term analysis of NP toxicity: rat L6 skeletal muscle cells and two types of normal porcine urothelial (NPU) cells. The NPU model presents the urothelial barrier expressing tight junctions and has a good transferability to animal and human studies [39,40,41,42,43], while L6 myoblasts can differentiate into post-mitotic myocytes and multinucleated myotubes [44]. The urothelial barrier model is appropriate for the testing of NPs that can be secreted into urine as in pathological cases, such as urine retention and incomplete urine voiding, they can persist for longer periods of time in contact with these cells [45]. Cells in such differentiated models do not divide, enabling us to observe the effects of intracellular NP accumulation, as would be expected for tissue accumulation. The myoblast/myotubes model represents a simple in vitro model of cells that are able to differentiate into metabolically very active muscle tissue [46,47]. Moreover, such models also allow us to study the effects of NPs on cell differentiation, which is an important regenerative process in muscle tissue.

We performed short-term (96 h) and long-term (10 days for L6 cells, 31 days for NPU cells) toxicity studies of two sets of NPs: (i) biomedically relevant magnetic NPs (Co–ferrite–polyacrylic acid (PAA) and Co–ferrite–polyethylenimine (PEI)) and (ii) two industrial NP types used in consumer products: SiO_2_ NPs used in cleaning solutions and TiO_2_ NPs used for coatings. The range of selected NPs concentrations was adopted to exposure protocol: we used very low concentrations that can be realistically achievable in vivo to simulate chronic exposure. To simulate acute exposure, we used moderate to high concentrations, which are typically used in various in vitro studies. We observed time and dose-dependent decrease in viability that were most pronounced for TiO_2_ NPs. In general, L6 cells were much more sensitive to NPs than NPU cells. The internalization of NPs was confirmed only for L6 cells, while in NPU cells, the internalization of NPs was not observed. The effects on L6 cells were analyzed in more detail by means of ROS induction, levels of nuclear factor (erythroid-derived 2)-like 2 (Nrf2), and effects on differentiation. Interestingly, TiO_2_ NPs affected the differentiation level of L6 cells as determined by decreased mRNA levels of myogenin (MyoG) and myosin I (MyhI) [48]. Altogether, the presented study introduces differentiating and differentiated in vitro cell models for the assessment of chronic toxicity of NPs, thus surpassing the drawbacks of a standard in vitro cell subcultivation.

## 2. Results

### 2.1. Nanoparticle Characterization

NP characterization (dynamic light scattering, DLS; zeta potential, ZP) was performed in distilled water and in the two cell culture media used to differentiate L6 and NPU cells, since the media composition affects the stability/size and ZP (zeta potential) of NPs [3,4]. The primary size and shapes of NPs in water are presented in Figure S1; it can be seen that PAA and PEI NPs form smaller aggregates (size of primary core is around 20 nm), while SiO_2_ and TiO_2_ NPs form larger aggregates (200–300 nm) in water (primary crystals are relatively small) in agreement with DLS. The pH of NPs suspensions at concentrations used for the DLS/ZP measurements in water were pH_PAA_ = 7.4, pH_PEI_ = 6.6, pH_SiO2_ = 2.9, and pH_TiO2_ = 4. The measurements of hydrodynamic diameters obtained from the number distribution, z-average size, and ZP (Table 1) demonstrated significantly different behavior of the tested NPs; the number-based size distributions (representative measurements) in all media are shown in Figure S2.

The measurement of PAA, PEI, and TiO_2_ NPs showed an increase in the hydrodynamic diameter of NPs in cell culture media due to additional layers of molecules with the opposite charge. Furthermore, macromolecules present in the media formed the so-called biocorona on the surface of NPs [4]. Please note that TiO_2_ has positive ZP in water due to acidic pH. For the same reason, the absolute values of ZP in general dropped when measured in the culture media. Interestingly, PDI values show that TiO_2_ and PEI NPs had more narrow size distributions in all media compared to PAA and SiO_2_ NPs.

SiO_2_ NPs were originally stabilized with citric acid and dispersed in ethanol at pH 2 with a size around 15 nm (data provided by the manufacturer). When dispersed in distilled water, the number-based peak of hydrodynamic diameter was 238 nm with a ZP of 2.4 mV. We have to stress that the DLS and ZP measurements of SiO_2_ in medium do not present the complete distribution of NPs, as most of the NPs aggregated immediately when re-suspended in the cell culture media [4], and therefore, only a smaller fraction of NPs was still dispersed. We have also previously shown that SiO_2_ NPs formed aggregates 1.5–100 μm in diameter at pH 7 [4]. It should be noted that the PDI was generally quite high in cell media and that suspensions of NPs were therefore polydispersed, as can be seen from (Figure S2). Importantly, a wide distribution of NPs hydrodynamic radii indicates that NPs suspensions in culture media are comprised of relatively small NPs, small aggregates, and also large aggregates (Figures S1 and S2), depending on the properties of each NP, as well as the characteristics and properties of the specific media.

### 2.2. The Characteristics of L6 and NPU Cells and Exposure Protocols for the Long-Term Studies

**Figure 1:**
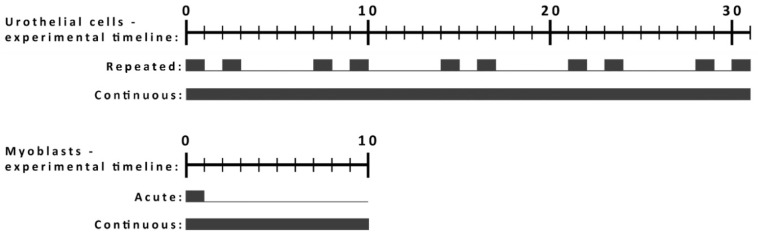
Schematic representation of the exposure protocols for the long-term studies. Long-term nanotoxicity studies were performed using normal porcine urothelial (NPU) cells and rat L6 cells. Cells were exposed to NPs as indicated in the scheme: a full line denotes NP exposure, while an empty line means cell culturing in the absence of NPs. NPU cells were grown for 31 days and exposed to NPs either 2 days per week (repeated) or continuously for 31 days (continuous). L6 cells were grown for 10 days and were exposed to NPs either only on the first day (acute) or for 10 days (continuous) during their differentiation.

Short-term and long-term toxicity were evaluated on two in vitro models. An NPU urothelial model was chosen as an advance 3D barrier model of terminally differentiated cells that resembles tissue properties, while a partially differentiated NPU model presents a case of injured urothelium. Differentiating L6 myoblasts were chosen as a more simple and easy to use model of a muscle tissue that has great regeneration properties. We used this model to analyze the potential effect of NP exposure on the differentiation of myoblasts into myotubes. The protocols of exposure were adapted for each model (Figure 1).

### 2.3. Short-Term Assessment of NPU and L6 Cell Viability for 96 h Exposure to NPs

**Figure 2:**
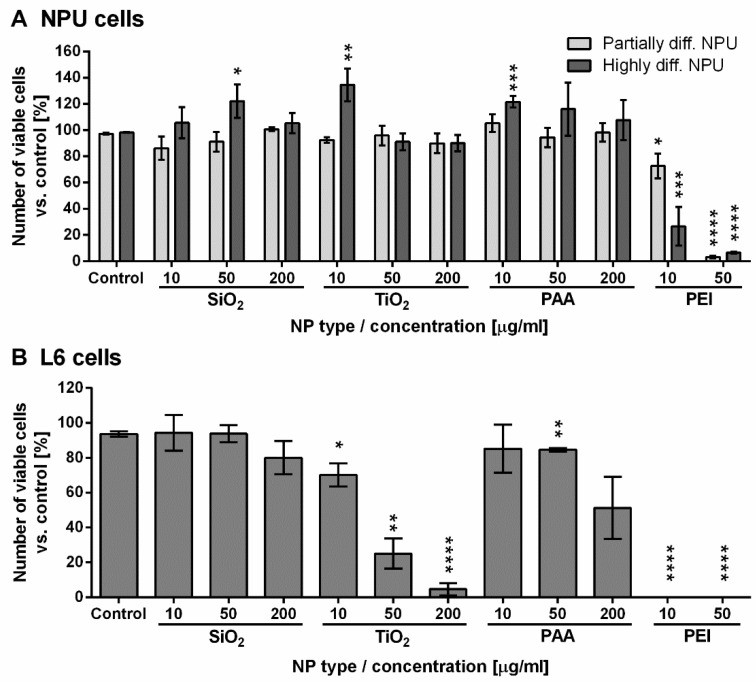
Short-term assessment of NPU and L6 cell viability for 96 h exposure to NPs. Viability of (**A**) highly and partially differentiated NPU cells and (**B**) L6 cells following 96 h of exposure to increasing concentrations of SiO_2_, TiO_2_, PAA, and PEI NPs. The results are presented as the percentage of viable cells compared to the number of all cells in the control sample for each cell type. Mean and standard error are shown. (Partially differentiated NPU; SiO_2_, PAA, and PEI n = 4; TiO_2_ *n* = 2; highly differentiated NPU; SiO_2_ 10 and 50 μg/mL, *n* = 2; SiO_2_ 200 μg/mL, *n* = 3; TiO_2_, *n* = 2; PAA NPs 10 μg/mL, *n* = 2; PAA NPs 50 and 200 μg/mL, *n* = 3; PEI, *n* = 2; L6 cells, *n* = 3; n stands for the number of independent experiments, each performed in three replicates). Statistical significance is displayed as follows: * *p* ≤ 0.05; ** *p* ≤ 0.01; *** *p* ≤ 0.001; **** *p* ≤ 0.0001.

We assessed the viability of NPU and L6 cells after 96 h exposure to NPs (Figure 2). Both types of NPU cells showed almost no reduction in cell viability after the treatment with different concentrations of SiO_2_, TiO_2_, or PAA NPs for 96 h (Figure 2A). However, a significant drop in cell viability was detected when these cells were treated with PEI NPs, which reduced cell viability to 72% (partially diff. NPU) and 26% (highly diff. NPU) for 10 µg/mL PEI NP and to approximately 5% viability for both cell models following incubation with 50 µg/mL PEI NPs (Figure 2A).

L6 cells were more susceptible to the presence of NPs (Figure 2B). While SiO_2_ and PAA NPs showed minor toxicity only at the highest 200 µg/mL concentrations (80% and 50% viability, respectively), TiO_2_ induced a strong dose-dependent cytotoxic effect, dropping to 5% viability at the highest dose. L6 cells did not survive the treatment with either concentration of PEI NPs (Figure 2B).

### 2.4. Long-Term Assessment of NPU and L6 Cell Viability for Exposure to NPs

To assess the long-term toxicity of SiO_2_, TiO_2_, and PAA NPs, exposure protocols were adapted to each cell model (Figure 1). For urothelial models of differentiated or partially differentiated NPU cells, we performed a 31-day experiment. NPU cells were exposed to NPs either for 2 days per week for 24 h (repeated exposure) or continuously for 31 days (continuous exposure) (Figure 1). Both protocols of exposure were used to simulate chronic exposure to NPs (e.g., NPs that are present in food) on an everyday basis (continuous exposure) or periodically (repeated exposure). The 31-day cultivation enabled us to observe potential cytotoxic effects due to long-term exposure.

To assess the toxicity of NPs on differentiating L6 cells, cells were grown for 10 days (9 days in differentiating media) during which L6 cells start to form myotubes. L6 cells were exposed to NPs either for 24 h followed by 9 days of culturing without NPs (acute exposure) or for all 10 days (continuous exposure). An exposure of 24 h was used as it is a commonly used acute exposure duration that simulates a single-event exposure to a specific NP, while continuous exposure protocols again simulated a constant exposure to a given NP on an everyday basis. For both protocols, 10-day experiments enabled us to study the effects also on the differentiation of L6 into myotubes.

**Figure 3:**
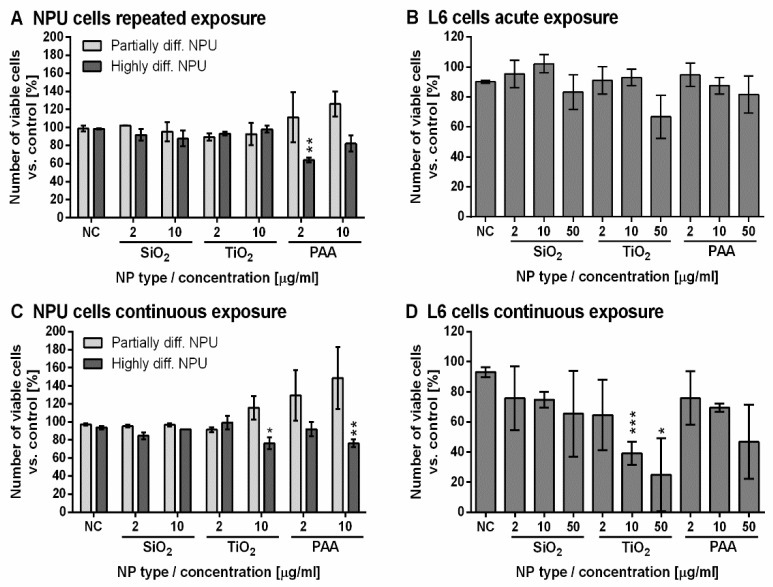
Long-term assessment of NPU and L6 cell viability for exposure to NPs. Long-term viability of (**A**) repeatedly exposed highly and partially differentiated NPU cells, (**B**) acutely exposed L6 cells, (**C**) continuously exposed highly and partially differentiated NPU cells and (**D**) L6 cells continuously exposed to increasing concentration of SiO_2_, TiO_2_, and PAA NPs. The results are presented as the percentage of viable cells compared with the number of all cells in the control sample for each cell type. Mean and SEM are shown. (Experiments on NPU cells were performed in *n* = 2 and experiments on L6 cells in *n* = 3; n stands for the number of independent experiments, each performed in three replicates). Statistical significance is displayed as follows: * *p* ≤ 0.05; ** *p* ≤ 0.01; *** *p* ≤ 0.001.

The results of long-term exposure studies (31 days for NPU or 10 days for L6 cells) clearly show that NPU cells were overall much more resistant against NP-induced damage compared to L6 cells. In the case of both types of NPU cell models, only TiO_2_ and PAA NPs at the highest tested concentrations showed a slight toxic effect (Figure 3A,C). The results of repeated and continuous exposure for NPU cells also show that highly differentiated NPU cells are slightly more sensitive to NPs compared with partially differentiated ones (Figure 3A,C).

For acute exposure, SiO_2_ and PAA NPs were not significantly toxic to L6 cells, while TiO_2_ showed a small (statistically not significant) cytotoxic effect at 50 µg/mL (Figure 3B). In the case of continuous exposure of L6 cells, there was a significant dose-dependent response. TiO_2_ NPs were the most toxic of all three types of NPs tested (Figure 3D), and already for low 2 µg/mL concentration, the viability was reduced to 65%. PAA and SiO_2_ induced only a small, not statistically significant decrease in viability at the highest concentration.

### 2.5. The Morphology of the L6 Cells Treated with NPs and Analyzed with Phase-Contrast Microscopy

**Figure 4:**
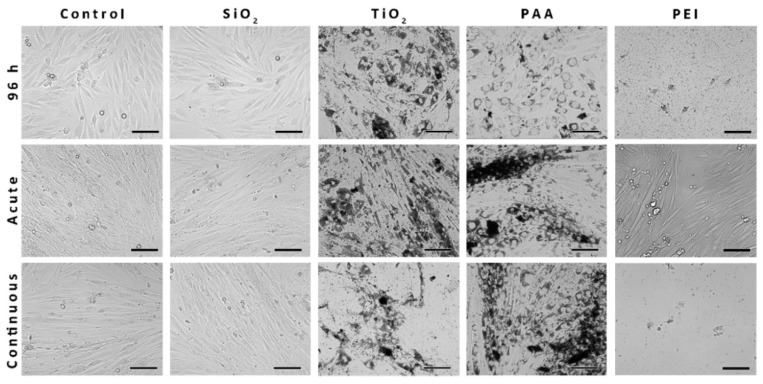
The morphology of the L6 cells treated with NPs and analyzed with phase-contrast microscopy. Cells were incubated with SiO_2_, TiO_2_, PAA (50 µg/mL), or PEI (10 µg/mL for 96 h experiment or 2 µg/mL for 10-day experiment) NPs either for 96 h or 10 days (acute and continuous exposure protocol). Scale bars correspond to 100 µm.

To visualize the uptake of NPs into L6 cells and determine if the observed intracellular retention of NPs affects L6 cells differentiation and morphology, phase-contrast microscopy was performed (Figure 4). While PAA, TiO_2_, and PEI NPs could be clearly seen under a phase-contrast microscope, the SiO_2_ NPs could not be observed. After 10 days of cultivation, L6 cells became elongated multinucleated cells and formed myotubes, as seen in the control sample. SiO_2_ NPs did not affect the morphology of the L6 cells, since 96-h L6 myoblasts or 10-day-old L6 myotubes appear morphologically very similar to the cells in the control samples. The uptake of TiO_2_ and PAA NPs observed after 96 h or 10 days of exposure significantly affected the morphology of the cells. PEI NPs were highly toxic, and only some non-differentiated live L6 cells could be found following acute 10-day incubation (Figure 4).

### 2.6. TEM Images of NPU Cells and L6 cells after Prolonged Exposure to NPs

**Figure 5:**
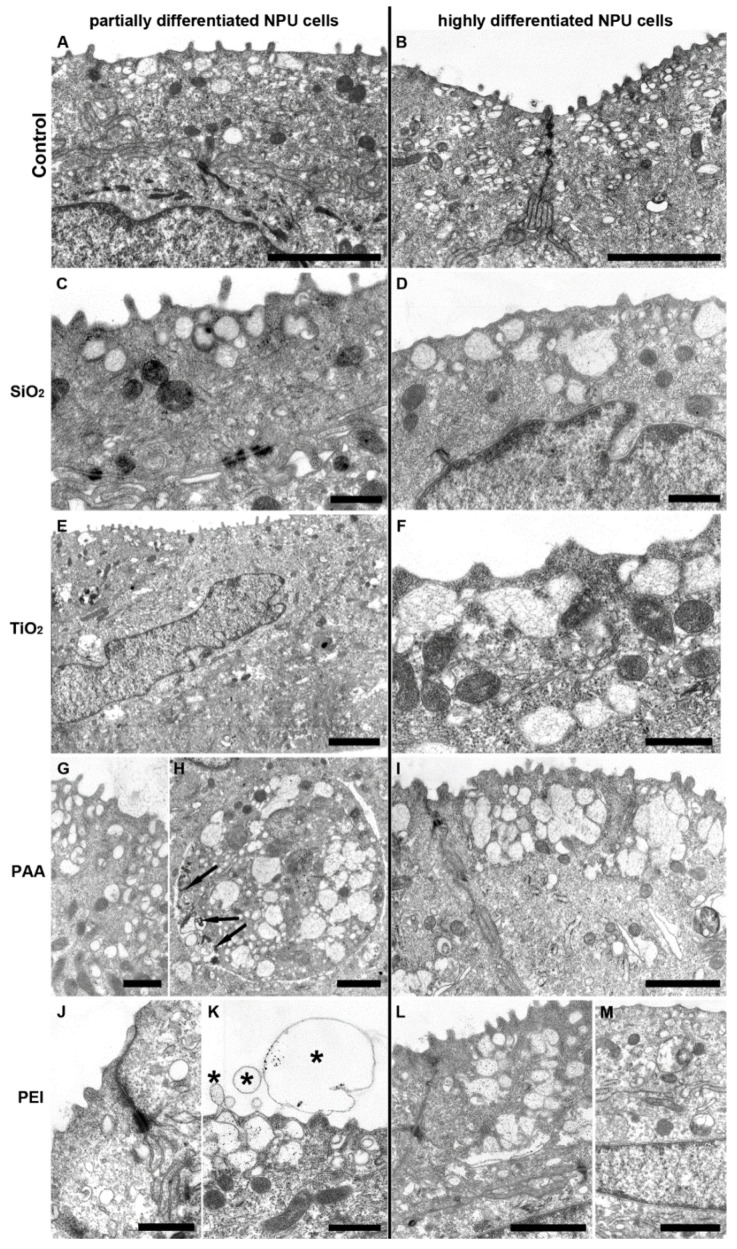
TEM images of partially differentiated and highly differentiated NPU cells after prolonged exposure to NPs. (**A**,**B**) Control, untreated NPU cells and NPU cells treated with (**C**,**D**) SiO_2_, (**E**,**F**) TiO_2_, (**G**,**H**,**I**) PAA (50 µg/mL), and (**J**–**M**) PEI (2 µg/mL) NPs for 31 days in continuous exposure experiment. In a partially differentiated NPU model, exfoliations of apical plasma membrane were found very rarely and only in PEI-treated cells (asterisks). Aggregates of the contrasting agent (arrows) and not PAA nanoparticles are seen in H. In NPU cells of partially and highly differentiated models, many fusiform vesicles are present in the apical cytoplasm and well-developed junctional complexes (tight junctions, adherent junctions, and desmosomes), which are specific morphological characteristics of urothelial cells, scale bars: 500 nm (**C**,**D**,**F**,**G**,**J**,**K**), 1 µm (**H**,**I**,**L**,**M**), and 2 µm (**A**,**B**,**E**).

Both partially and highly differentiated NPU cells were treated with SiO_2_, TiO_2_, PAA (50 µg/mL), and PEI coated NPs (2 µg/mL) with a Co ferrite core for 31 days in a continuous exposure experiment (Figure 5). Despite an extensive search for endocytosed NPs, no particles were found in NPU cells (Figure 5A–M). In addition, no morphological changes of these cells were detected compared to cells in the control experiment, except in PEI-treated cells where higher concentrations of NPs induced some exfoliations of the apical plasma membrane (Figure 5K).

**Figure 6:**
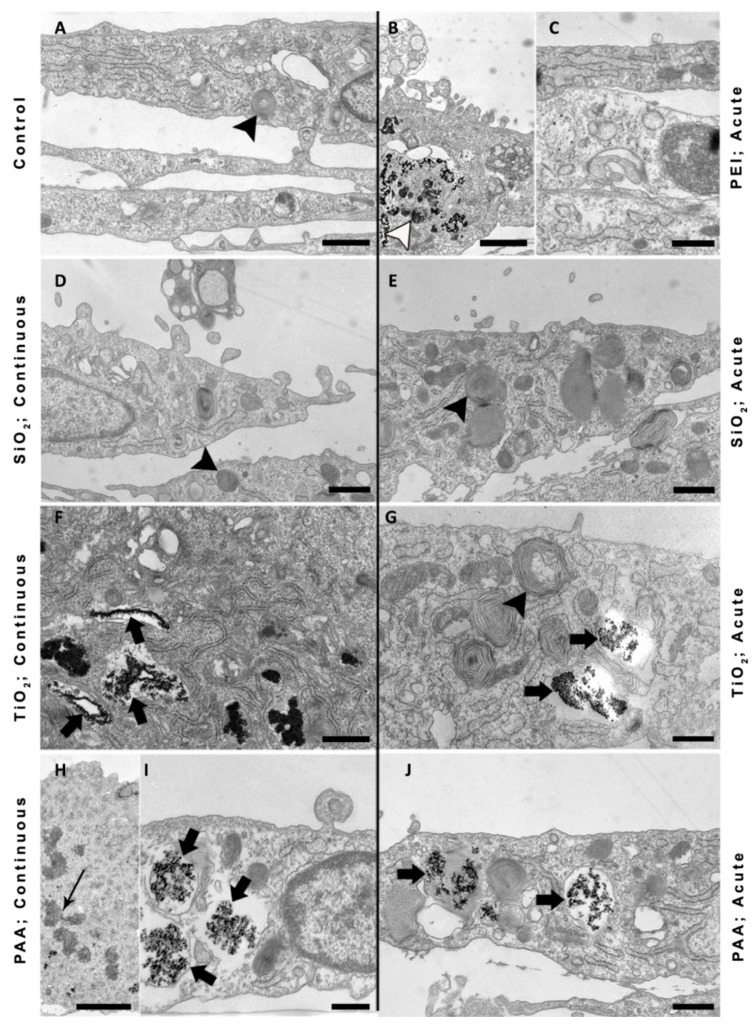
TEM images of L6 cells after prolonged exposure to NPs. Cells were exposed to (**A**) control; (**B**,**C**) PEI (2 µg/mL) NPs, (acute exposure); (**D**,**E**) PAA NPs (continuous exposure); (**F**) PAA (acute exposure); (**G**), SiO_2_ (continuous exposure); (**H**) SiO_2_ (acute exposure); (**I**) TiO_2_ (continuous exposure); (**J**) TiO_2_ (acute exposure). Black arrowheads point to multilamellar bodies, open arrowhead points to amphisomes, long arrow points to chromosomes of the mitotic cell, short arrows point to endolysosomal compartments. Scale bars: 20 μm.

Furthermore, we treated L6 cells with SiO_2_, TiO_2_, PAA (50 µg/mL), and PEI (2 µg/mL) for 10 days either in an acute or in a continuous mode of exposure and acquired images by TEM (Figure 6). We can notice several multilamellar bodies within the L6 control as well as within treated cells (Figure 6; black arrowheads). L6 cells exposed to TiO_2_, PAA, and PEI NPs in the acute experiment are still loaded with NPs, indicating that these NPs are not exocytosed, as seen also in phase-contrast images. The ultrastructure of these cells looks normal with the exception of PEI-treated cells, where we noticed vesicles with heterogeneous content likely to belong to the endosomal autophagic category of amphisomes (Figure 6B; open arrow). L6 cells accumulated TiO_2_ and PAA NPs in endolysosomal compartments (Figure 6F,G,I,J; black arrow). SiO_2_-treated L6 cells looked very similar to the control cells in both exposure protocols and no internalization could be observed, which was probably due to the extensive aggregation (aggregate > 3µm) of SiO_2_ NPs_._

The distribution of NPs and the morphology of the cells were relatively similar also in continuously exposed L6 cells. All internalized NPs were found aggregated in endocytotic compartments within the cell cytoplasm and no NPs were found free in the cytosol or associated with other intracellular organelles. The endosomes and other intracellular organelles of NP-treated cells were comparable in size and structure to control cells. Additionally, we found mitotic L6 cells with endocytosed PAA NPs, indicating cell proliferation (Figure 6H; arrow).

### 2.7. TiO_2_, PAA, and SiO_2_ NPs Induced ROS Formation in L6 Cells in a Dose-Dependent Manner

**Figure 7:**
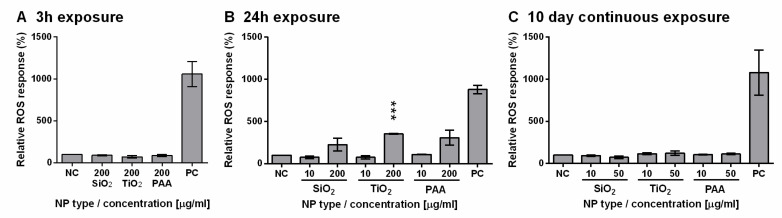
TiO_2_, PAA, and SiO_2_ NPs induced reactive oxygen species (ROS) formation in L6 cells in a dose-dependent manner. ROS induction after (**A**) 3 h, (**B**) 24 h, and (**C**) 10-day exposure of rat L6 cells to SiO_2_, TiO_2_, or PAA NPs (10, 50 or 200 µg/mL). Results are shown as increased compared to the control, which was not exposed to NPs. Mean and standard error are shown (3 h, *n* = 2; 24 h, *n* = 2, 10 days, *n* = 3; n stands for the number of independent experiments, each performed in 3 replicates; PC stands for positive control, 1 mM H_2_O_2_). Statistical significance is displayed as follows: *** *p* ≤ 0.001.

Oxidative stress was determined by measuring relative ROS levels in L6 cells exposed to three types of NPs (SiO_2_, TiO_2_, and PAA). Cells were exposed to NPs for 3 h, 24 h, or for 10 days in continuous exposure protocol (Figure 7). There was no significant increase in ROS levels for 3 h or 10-day exposure protocols. However, all three types of NPs induced ROS formation in a dose-dependent manner after 24 h of incubation. A lower concentration of NPs (10 µg/mL) did not induce ROS formation, while at high concentrations (200 µg/mL) of TiO_2_, PAA, and SiO_2_, NPs induced a significant increase in ROS levels compared to the control cells. After 10 days of continuous exposure, ROS levels were close to basal levels for 10 and 50 µg/mL.

### 2.8. The Influence of TiO_2_, PAA, and SiO_2_ NPs on L6 Cells Differentiation and Oxidative Stress Biomarkers

**Figure 8:**
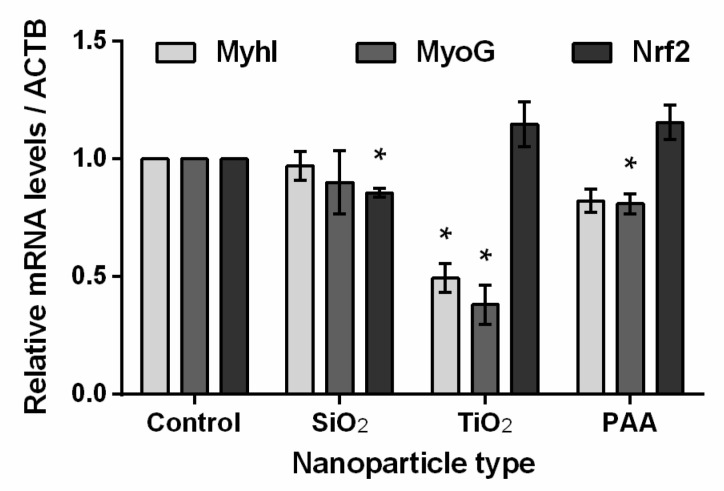
The influence of TiO_2_, PAA, and SiO_2_ NPs on L6 cells differentiation and oxidative stress biomarkers. Fold change in gene expression of myosin I (MyhI), myogenin (MyoG), and nuclear factor (erythroid-derived 2)-like 2 (Nrf2), after treating differentiating rat L6 cells for 10 consecutive days with SiO_2_, TiO_2_, and PAA NPs (50 µg/mL). Results are normalized to Actin beta (ACTB) and shown as a fold increase compared to the non-treated control. Means with standard error are shown for three independent experiments, each performed in three replicates. Statistical significance is displayed as follows: * *p* ≤ 0.05.

The effects of NPs on oxidative stress and cell differentiation were analyzed also on a molecular level using qPCR. Differentiating rat L6 cells were treated for 10 days (continuous exposure) with 50 µg/mL SiO_2_, TiO_2_, and PAA NPs. The quantity of myosin I, myogenin, and Nrf2 mRNA transcripts was determined and normalized to ACTB mRNA expression levels (Figure 8). The treatment of L6 cells with SiO_2_ NPs did not affect the expression of MyhI and MyoG, while the expression of Nrf2 decreased by 15%. TiO_2_ NPs negatively affected the differentiation of L6 cells (the expression levels of MyhI and MyoG mRNA were decreased by 50% and 60%, respectively), while the expression of the Nrf2 gene was increased by approximately 15% (not statistically significant). The influence of PAA NPs on L6 differentiation was less pronounced (20% decrease) compared to the effect of TiO_2_. The effect of PAA NPs on the mRNA expression level of Nrf2 was comparable to TiO_2_ NPs (relative increase by 15%).

## 3. Discussion

The presence of NPs in the environment and the potential toxicity of some engineered NPs such as TiO_2_ and SiO_2_ that are used in many everyday products still present unresolved health dilemmas despite many in vitro and in vivo studies done so far [1,49,50,51]. While short-term and acute exposure experiments are routinely performed, there are not many suitable protocols for the assessment of long-term cytotoxic effects of NPs in vitro. Moreover, there is a lack of long-term nanotoxicity studies that would test also lower, physiologically more relevant concentrations of NPs as stressed by several regulating bodies [52,53,54,55]. Caution is especially needed in the case of non-degradable NPs that can accumulate in the organs and body fluids after repeated exposure. Therefore, the main goal of our study was to implement more advanced biomimetic in vitro models to assess the short-term and long-term effects of exposure to the selected types of non-degradable NPs in order to bypass cells’ subcultivation in vitro and to follow the “three R” strategy to reduce the use of laboratory animals. In this study, we implemented two in vitro cell models: (a) differentiating the L6 rat skeletal muscle cell line, and (b) an urothelial in vitro model of partially differentiated NPU and highly differentiated NPUs. Both in vitro models enabled us to analyze the effects of NPs on (i) already differentiated NPU cells (typical somatic cells) or (ii) on differentiation (L6 myoblasts/myotubes) and could be used for the testing of NPs that cross the barriers into the blood and with transcytosis through endothelial cells being deposited in the muscle tissue and/or secreted through the kidneys into the urine.

We evaluated the cytotoxicity for short-term (96 h) and long-term (10 days for L6 cells and 31 days for NPU cells) exposure experiments, where the long-term exposure to NPs was performed either as continuous (for both cell types), repeated (NPU cell), or acute exposure (L6 cells) (Figure 1). Acute exposure simulated exposure to a single event, such as the administration of biomedical NPs (e.g., exposure to NPs used as contrast agents for NMR imaging) or an acute single exposure to industrial NPs. A continuous exposure simulated events of repeated or continuous exposure to NPs due to the presence of NPs in different consumer products or environmental pollution. We used four types of NPs: two biomedically relevant (magnetic PAA and PEI) NPs [8,56,57,58,59] and industrially relevant (SiO_2_ and TiO_2_) NPs as representative types of two very commonly used commercial NPs in various consumer products and materials. We analyzed the effects of NPs on the cell viability, morphology, and ultrastructure on both cell models. Furthermore, the effects of NPs on the stress response and differentiation of L6 cells were analyzed.

The physiochemical properties of NPs’ zeta potential, hydrodynamic radius, aggregation, and surface-bound molecules are highly determined by the presence of ions and other charged molecules in biological media. Since these physiochemical properties of NPs significantly affect physiological interactions between nanomaterials and target biological areas [57], it is crucial that for each NPs type, measurements of physiochemical properties are performed in the physiologically relevant media [4]. Therefore, DLS and zeta potential (ZP) measurements were performed both in water and in the relevant culture media.

Magnetic PAA NPs are negatively charged and highly stable in physiological conditions, but they are less stable at high calcium concentration (Table 1) [3]. As already described, they are short-term nontoxic and can be internalized in high quantities [56,60]. On the other hand, due to their highly positive zeta potential, [61,62] magnetic PEI NPs aggregated, and their size increased up to 10 times when dissolved in cell culture media due to the formation of biocorona and aggregation [4,62]. Due to their positive zeta potential, PEI NPs bind strongly to negatively charged cell membranes, which is one of the main mechanisms of their high toxicity [58,62]. TiO_2_ NPs partially retained their stability in the cell culture media despite the change in ZP [63], while DLS and ZP measurements of SiO_2_ NPs should be discussed with caution due to extensive aggregation (see results), which does not allow accurate measurements [62]. Extensive aggregation also hindered the internalization of these NPs, which further demonstrates the importance of characterization also in physiological conditions. The aggregation of NPs in physiological media can significantly limit their mobility. Therefore, their ability to cross the biological barriers and cellular uptake can be reduced, which can in turn importantly affect potential toxicity [62].

If we summarize the results of short-term (96 h) and long-term (31 day) viability analysis on NPU cells, it can be observed that the PAA, TiO_2_, and SiO_2_ NPs used in the study were in general not toxic for NPU cells. For short-term exposure, only PEI NPs induced significant toxicity on NPU cells after 96 h in the chosen range of concentrations (10–200 µg/mL), which was most probably through membrane damage [62]. Long-term (31 day) viability analysis on NPU cells further demonstrated only small toxicity for the continuous exposure for PAA and TiO_2_ NPs at 10 µg/mL concentration.

On the other hand, L6 cells were much more susceptible to the presence of NPs, and a concentration-dependent decrease in cell viability could be observed for all used NP types (magnetic PAA, magnetic PEI, TiO_2_) except SiO_2_ (Figure 2 and Figure 3).

The differences in susceptibility to NPs exposure between both cell models can be explained with the specific properties of each model. L6 cells grow in a monolayer, while NPU cells grow in several layers, and when differentiated, they form a tight barrier, which is impermeable to most substances [64,65]. Moreover, both NPU cell models have been shown to have an extremely low endocytic rate [65], which can result in a lack of NP internalization [66], as observed by TEM (Figure 5). Thus, the barrier function of NPU cells and low level of endocytosis is probably the main reason for large differences in viability compared to L6 cells. In addition, NPU cells grow in multiple layers; thus, in an NPU cell model, the superficial layer of cells is the most exposed to NPs, while in a monolayer L6 cell culture, all the cells can be in direct contact with NPs.

In L6 cells, dose and time-dependent effects on cell viability were observed depending on the physiochemical properties of NPs. In general, aggregated industrial SiO_2_ induced small toxic effects only at extremely high concentration, while relatively stable TiO_2_ and magnetic PAA NPs that could be internalized had several effects also at moderate concentrations. The short-term exposure (96 h) of L6 cells to TiO_2_ induced a significant decrease in viability, and the highest concentration of PAA NPs induced a drop in L6 cell viability (statistically not significant) similarly as already observed on primary human myoblasts [56]. The drop in viability was probably due to membrane damage in case of magnetic PEI NPs, while for other NPs, there is little effect due to membrane damage (low percentage of PI positive cell), and effects are mostly on cell proliferation as already shown for magnetic PAA [56,67].

Interestingly, the long-term cytotoxicity of SiO_2_, TiO_2_, and magnetic PAA on L6 cells was observed already at moderate NPs concentrations (10 µg/mL), and a decrease in viability (but statistically not significant) for TiO_2_ was observed even for relatively low (2 µg/mL) NPs concentrations. Here, we have to stress that for all types of exposure, NPs have to cross one or several biological barriers; therefore, we expect the concentrations to be lower then 2 µg/mL. On the other hand, inorganic NPs can persist in cells for a long time and can accumulate; therefore, 2 µg/mL can be realistically achieved in tissues.

Overall, several studies of long-term effects of TiO_2_ NPs were already performed, and they show conflicting results. The discrepancy between different results is not surprising, as diverse formulations of TiO_2_ NPs with different physiochemical characteristics exist. Wang et al. found no reduction of cell viability or cell division rate when they exposed CHO cells (60 days of subcultivation) to anatase TiO_2_ (0–40 µg/mL, 25 nm) NPs [38]. Similar viability results were obtained by Brun et al., who applied a single dose of 75% anatase TiO_2_ NPs (1–200 µg/mL, 25 nm) to an in vitro rat primary cell based blood–brain barrier model in either acute (24 h) or chronic (5 days) exposure [19]. They also showed that chronic exposure alters blood–brain barrier integrity and function and modulates the inflammatory response. Anatase TiO_2_ NPs (15 nm) were also shown to affect the proliferation of human astrocyte and neuronal cells in vitro even at low concentrations (0.05–31 µg/mL) following 7–10 days of continuous exposure [22].

In general, herein, used industrial SiO_2_ NPs did not show any substantial toxic effect and were the least toxic of all tested NPs for both cell types. This is in agreement with the observed larger aggregation of NPs in physiological media that hinders NPs internalization. More generally, amorphous SiO_2_ NPs were shown in many studies to be nontoxic [68], but due to many different types of SiO_2_ NPs, their toxicity depends upon the physicochemical characteristics of the particles [59,69], and the response against SiO_2_ NPs varies in cell-dependent manner [70,71,72]. There are not many long-term studies examining the effect of SiO_2_ NPs. For example, Qignard et al. incubated human dermal fibroblasts with various types of fluorescent SiO_2_ NPs for up to 14 days [33]. They found that the tested formulations of SiO_2_ NPs are not toxic per se and are strongly dependent on their size and zeta potential. Similarly, in the present study, the tested SiO_2_ were generally nontoxic and could probably not undergo internalization due to extensive aggregation in the culture media.

To analyze the effects of long-term NPs exposure on the cell morphology, ultrastructure, and NP intracellular fate, phase-contrast and electron microscopy were performed. In healthy cells of both highly and partially differentiated NPU cells, no internalized NPs were detected, which is consistent with previous observations that NPU cell models exhibit no endocytic activity (Figure 5) [66,73]. Interestingly, while PEI NPs showed moderate toxicity to NPU cells, no internalized NPs were observed, indicating that PEI NP-induced plasma membrane damage, which was also found by TEM (Figure 5).

On the other hand, differentiating L6 cells internalized and retained TiO_2_ and PAA NPs in high quantities (Figure 6) even 9 days after 24 h acute exposure, demonstrating that NPs can be present inside the cell for a prolonged period of time. Again, no internalization of large SiO_2_ aggregates could be observed under TEM, which was most probably due to their large size (Figure 6D,E). All internalized NPs were enclosed in endosomes, and no NPs were found free in the cytosol. We did not observe PEI NPs in endosomal compartments, and most of the PEI damage can be attributed to its toxicity on the membrane [58,62]. TEM results suggest that a high level of endocytosis of NPs is the main reason for decreased viability in L6 cells for PAA and TiO_2_ compared to non-endocytotic NPU cells. Cell types with lower endocytotic potential are less affected by the presence of NPs, while in the cell types that internalize more NPs, the toxic effects are more pronounced, since internalized NPs can directly affect cellular processes and trigger other toxicity mechanisms related to the degradation of NPs. Thus, the differences in the endocytic potential and barrier functions of some cell types suggest which cell types and tissues within the human body would be most affected in case of systemic exposure to NPs.

As a significant reduction in viability occurred only in L6 cells, we therefore focused on these cells for further analysis of the mechanisms of observed NP toxicity. Since ROS generation and oxidative stress are very often detected along with NP-associated toxic events [74,75] such as genotoxicity, inflammation, fibrosis, and carcinogenesis [76], we analyzed intracellular ROS levels in an L6 cell model. We observed a transient increase in levels of ROS after 24 h of exposure at the highest concentrations (200 μg/mL) of PAA, TiO_2_, and SiO_2_ NPs, while no increase in ROS levels was observed after 3 h or 10 days of exposure. This could be explained with a feedback response, which is in agreement with the observed increased levels of Nrf2 mRNA for PAA and TiO_2_ NPs after continuous 10-day exposure, as determined with qPCR (Figure 8). Increased Nrf2 after 10-day exposure indicates a still active cell stress response against oxidative stress, which might explain the observed increase in ROS at 24 h after incubation. This is also in agreement with Wang et al., who showed that ROS increased in a concentration-dependent manner and that long-term cultures (60 days) had lower levels of oxidative stress compared to the short-term exposures (2 days) to TiO_2_ NPs [38].

The transient increase in ROS levels and activation of Nrf2 transcription factor for TiO_2_ and to a lesser extent PAA correlates with the highest cytotoxicity of these two NPs and is in agreement with other studies, where it has been shown that TiO_2_ and iron oxide NPs can induce apoptosis and other changes in vitro through ROS-mediated mechanisms [73,77,78,79,80,81,82,83,84,85]. Furthermore, mild oxidative stress activates phase II antioxidant enzymes through the activation of Nrf2 transcription factor, while high levels of ROS overwhelm a cell’s protective mechanisms and cause cell dysfunction and cell death [75]. Nrf2 transcription factor is a central factor involved in stress response and inflammation-related signaling. Therefore, increased levels of Nrf2 mRNA for continuous 10-day exposure (50 µg/mL) to PAA and especially TiO_2_ indicate stress response and inflammation due to the continuous presence of NPs.

Furthermore, qPCR revealed that continuous 10-day exposure to TiO_2_ NPs (50 µg/mL) significantly decreased mRNA levels of MyoG and MyhI [48], which is in agreement with observed morphological changes of myotubes compared to the control sample (Figure 6) [86]. Most probably, a high concentration of internalized NPs that remain inside the cells directly disturbed normal cytoskeletal reorganization and thus hindered normal cell proliferation and differentiation processes [87]. Furthermore, increased ROS levels and oxidative stress, as a direct or indirect consequence of the presence of the NPs, can influence normal cell differentiation [88]. However, it is important to stress that 50 µg/mL concentrations of NPs or higher are difficult to achieve in vivo, except for in cases of chronic exposure and accumulation of NPs. Therefore, further experiments are needed to assess potential stress and immune responses.

## 4. Materials and Methods

### 4.1. Nanoparticles Synthesis

Magnetic PAA and PEI NPs were prepared as described previously [49,56,57]. Briefly, Co–ferrite NPs cores were prepared by a co-precipitation method [89] and stabilized in water. NPs (3 *w*/*w* %) were coated in situ with (3 *w*/*w* %) water solution of polyacrylic acid (PAA) sodium salt with molecular weight of 8 kDa (Sigma-Aldrich, St. Louis, MO, USA). Polyethylenimine (PEI) functionalization of NPs was performed using branched PEI (3 wt % water solution with a molecular weight of 25 kDa; Sigma-Aldrich, St. Louis, MO USA). Industrial TiO_2_ and SiO_2_ NPs were purchased from Nanotesla Institute (Ljubljana, Slovenia). SiO_2_ NPs were prepared by the sol–gel method and dispersed in a solution of citric acid in ethanol at pH 2. TiO_2_ NPs (Anatase) were prepared by the precipitation method with subsequent dry powder temperature processing and high-energy milling, while powder was re-suspended in acidic suspension.

### 4.2. Nanoparticles Characterization

For the hydrodynamic size characterization of the NPs, we used dynamic light scattering (DLS) (Malvern Zetasizer Nano ZS; Malvern Industries, Malvern, UK) with the NIBS (noninvasive backscatter) 173° backscatter algorithm. We are reporting the hydrodynamic diameter and polydispersity index (PDI). ZP (ξ potential) measurements were done with disposable folded capillary cells and the M3-PALS measurements technology built in a Malvern Nanosizer Nano ZS system. The size distribution of the NPs used in this study was evaluated in distilled water, in the cell culture medium used to differentiate L6 cells (MEM alpha + 2% FCS), and in UroM (+Ca-S_FBS_) growth medium with a high concentration of Ca^2+^ (2.5 mM Ca^2+^, no FBS), which was used to differentiate NPU cells. For the measurements of size distributions and ZP, the NPs’ original stocks were first vortexed and dispersed before the measurements in either distilled water or cell culture media at the following concentrations: 0.05 *w*/*w* % for PAA, PEI, and SiO2 NPs, and 0.02 *w*/*w* % for TiO2 NPs; the NPs suspension was re-suspended several times with the pipet. The pH of the NPs suspension in water or cell media was also measured at the used concentrations.

### 4.3. Cell Models and Cell Culturing

An L6 rat skeletal muscle cell line was obtained from ATCC. It was grown in minimal essential medium (MEM) alpha (Gibco, Invitrogen, Carlsbad, CA, USA), supplemented with 10% fetal calf serum (FCS) (Sigma-Aldrich, St. Louis, MO, USA), 1% PenStrep (100 units/mL penicillin and 100 mg/mL streptomycin) (PAA, Pasching, Austria), and 1% Fungizone (Gibco, Invitrogen, Carlsbad, CA, USA) at 37 °C in humidified air and 5% CO_2_. They were subcultivated every 2–3 days. Passage numbers 7–9 (counted from the vial received from ATCC) were used for the experiments. For the experiment, the cells were seeded in triplicate in 24-well plates at the seeding density of 1 × 10^4^ cells/well (short-term exposure) or 7 × 10^3^ cells/well (long-term exposure) and grown for 24 h in cell media supplemented with 10% FCS. After 24 h, the cell culture medium was exchanged for differentiating media (MEM alpha supplemented with 2% FCS, 1% PenStrep, and 1% Fungizone) that is used for L6 myoblasts cell differentiation into myotubes, and the cells were grown for an additional 96 h (short-term experiment) or 10 days in the presence of NPs (long-term experiment).

Normal porcine urinary bladders were obtained from the local slaughterhouse where the animals were inspected by the Veterinary Administrator of the Slovenian Ministry of Agriculture, Forestry, and Food. The bladders were handled aseptically and immediately immersed in a UroM medium adapted for the growth of NPU cells and handled as already described [39,42,90]. Before the experiments, all cultured cells were tested against mycoplasma DNA (PCR analysis) at the Institute of Microbiology and Immunology, Faculty of Medicine, University of Ljubljana. All cells were mycoplasma negative. For cell viability analysis, two types of urothelial cell cultures were used. For the establishment of highly differentiated culture, NPU cells were grown in UroM (+Ca^2+^ − S_FBS_), i.e., UroM cell culture medium without FBS, and supplemented with the physiological concentration of Ca^2+^ (2.5 mM) [40]. Partially differentiated NPU cells were maintained in the UroM (−Ca^2+^ + S_FBS_)—i.e., UroM medium with lower Ca^2+^ concentration (0.9 mM) and 2.5% FBS (Gibco, Invitrogen, Carlsbad, CA, USA). The cells were seeded in duplicate in 6-well plates (Corning Inc, Corning, NY, USA) with a seeding density of 1 × 10^5^ cell/cm^2^ and grown for 4 weeks before exposure to NPs. All NP incubation experiments were performed in the same culture medium as used for the establishment/differentiation of urothelial cell models.

### 4.4. Short-Term and Long-Term Exposure Protocols

Long-term toxicological studies were performed on highly differentiated and partially differentiated NPU cells and on differentiating rat L6 cell models. For both cell models, 96 h exposure to NPs as a short-term exposure was performed. Long-term exposure protocols were adapted for each cell model (Figure 1), since NPU and L6 cells differ regarding the time needed for differentiation. Moreover, in the case of NPU cells, we tested the long-term nanotoxicity of already differentiated cells, while in the case of L6 myoblast cells, we analyzed effects on differentiation; therefore, protocols were accordingly adapted. For NPU cells, we had two types of long-term experiments that both lasted for 31 days. In the first type, NPU cells were exposed to NPs for 2 days per week for 24 h: Repeated exposure. In the second type, NPU cells were exposed to NPs continuously: Continuous exposure (Figure 1). The cells were washed twice with phosphate buffered saline (PBS) before every addition of fresh cell culture media.

For L6 cells, we had two types of long-term exposures where we have tested the effects of NPs on the differentiation of L6 cells. Since L6 myoblast cells differentiate into myotubes in 10 days, that was the length of the acute and continuous exposure experiment. L6 cells were grown for 10 days in differentiating media and exposed to NPs either only on the first day, followed by 9 days of culturing without NPs (acute exposure), or for all 10 days (continuous exposure). Following NP incubation, cells were washed twice with PBS to remove non-internalized NPs and grown in fresh cell culture medium containing the appropriate NP concentration or in NP-free cell culture medium (Figure 1). The NPs concentrations used were 10, 50, and 200 µg/mL for short-term and 2, 10, and 50 µg/mL for long-term viability assays. The highest NPs concentrations were not used for PEI NPs due to their high toxicity.

Before the application to cells, the original stocks of NPs dispersions were vortexed; then, a specific quantity of NPs was dispersed in the cell culture media for L6 cells (MEM alpha + 2% FCS) or for urothelial cells (UroM − Ca^2+^ + S_FBS_) at the specific final concentration of NPs. The prepared NPs suspensions were re-suspended several times with the pipet.

### 4.5. Cell Viability Assays

Hoechst/propidium iodide (PI) viability assay was used to determine the viability of L6 cells [60,67]. Following NPs exposure, cells were washed and labeled with Hoechst 33,342 (2 μg/mL, 15 min) (Thermo Fisher Scientific, US; Life Technologies, USA) and PI (0.15 mM, 5 min) (Sigma, St. Louis, USA) and analyzed using Zeiss 200 (Axiovert, Oberkochen, Germany). Phase-contrast and at least 20 fluorescent images at 10× objective magnification were recorded for each fluorescent dye. Cells (stained nuclei) were counted using CellCounter software [60]. Thus, cell viability was estimated based on the number of Hoechst-positive (all) cells and PI-positive (dead) cells detected in 20 random fluorescent images. The number of viable cells for each sample was obtained by subtracting the number of PI-positive (dead) cells (N_PI_) from Hoechst-positive (N_H_) cells. The percentage of viable cells (% Viability) in a given sample was determined as the ratio between the number of viable cells in the sample (N_S_) and the number of all cells (Hoechst-positive) in the non-treated control N_H_ (control) [56,67].

N_s_ (Number of viable cells) = N_H_ (Hoechst-positive) − N_PI_ (PI-positive) (1)

(2) $$\%Viability=\frac{N_{S}}{N_{H}\left(control\right)}\times 100$$

As NPU cells grow in layers, trypan blue exclusion assay was used to determine the viability of these cell models. Following NP exposure, cells were washed and trypsinized using Triple Select (Gibco, ThermoFisher, Waltham, MA, USA) for 45 min. Then, trypsinized cells were mixed with Trypan Blue (ThermoFisher, Waltham, MA, USA USA) and used to selectively label dead cells, and all cells (N_ALL_) and dead cells (N_TB_) were counted using a Countess (Automated cell counter, ThermoFisher, Waltham, MA, USA, USA) with a size selection setting of 10–24 µm. The percentage of viable cells (% viability) in a given sample was determined as the ratio between the number of viable cells in the sample (N_S_) and the number of all cells in the non-treated control (N_KALL_), where

Ns = N_ALL_ (all cells) − N_TB_ (trypan blue positive cells) (3)

(4) $$\%Viability=\frac{N_{S}}{N_{KALL}\left(control\right)}\times 100.$$

### 4.6. Transmission Electron Microscopy

TEM was performed for NPU cells following continuous exposure and for L6 cells following acute and continuous exposure to 50 µg/mL of SiO_2_, TiO_2_, and PAA NPs and 2 µg/mL of PEI NPs. The control cells were incubated only in the cell culture medium. The cells were washed with PBS and fixed with a mixture of 4% (*w*/*w*) formaldehyde and 2% (*w*/*w*) glutaraldehyde in 0.1 M cacodylate buffer (pH 7.4) for 2 h at room temperature. Post fixation was carried out in 1% OsO_4_ in 0.1 M cacodylate buffer for 2 h, followed by dehydration in graded ethanol and embedding in Epon 812 resin (Electron Microscopy Sciences, Hatfield, PA, USA). Ultrathin sections were counterstained with uranyl acetate and lead citrate and examined with TEM (CM100; Royal Philips Electronics, Amsterdam, The Netherlands).

For additional TEM analysis, NPs suspensions (from the original stock) were re-suspended in 100 µL of distilled water with the following final concentrations: PAA, 200 µg/mL; PEI 200, µg/mL; TiO_2_, 100 µg/mL, and SiO_2_, 40 µg/mL. Then, 5 µL of this suspension was added on a copper grid covered with a formwar foil. After drying at room temperature, the samples were observed with a Philips transmission electron microscope CM100 TEM (Figure S1).

### 4.7. Measurements of the Intracellular ROS Levels in Rat L6 Cells

L6 cells were seeded in 24-well plates at 1 × 10^5^ cells/well for short-term experiments (3-h and 24-h incubation) and at 7 × 10^3^ cells/well for long-term experiments (10-day continuous exposure). For 3-h exposure, we used 200 µg/mL NPs; for 24-h exposure, we used 10 and 200 µg/mL; and for 10-day exposure, we used 10 and 50 µg/mL NPs. Following incubation, the cells were washed with PBS and incubated with 10 μM 5-(and-6)-chloromethyl-2′,7′-dichlorodihydrofluorescein diacetate (CM-H_2_DCFH-DA; Molecular Probes, Invitrogen, Carlsbad, CA, USA) in PBS (containing Ca^2+^ and Mg^2+^ ions) for 45 min at 37 °C. Fluorescence intensity was measured with excitation at 492 nm and emission at 527 nm using Tecan Infinite 200 (Tecan, Männedorf, Switzerland). As a positive control, 1 mM H_2_O_2_ was used. The fluorescence intensity of CM-H_2_DCFDA was normalized to the number of cells determined by fluorescence intensity of the Hoechst signal measured with excitation at 350 nm and emission at 461 nm using a Tecan Infinite 200 (Tecan Group Ltd., Männedorf, Switzerland).

### 4.8. Quantitative PCR

L6 cells were seeded in 24-well plates and continuously exposed to 50 µg/mL SiO_2_, TiO_2_, and PAA NPs for 10 days. The control cells were incubated only in the cell culture medium. Total RNA was extracted using RNeasy Mini Plus kit (Qiagen, Hilden, Germany) and reverse transcribed with High-Capacity cDNA Reverse Transcription kit (Applied Biosystems, Thermo Fisher Scientific, USA). Quantitative polymerase chain reaction (qPCR) was performed on an ABI Prism SDS 7500 sequence detection system (Applied Biosystems) using TaqMan chemistry in a 96-well format. We used TaqMan Universal PCR Master Mix and the following Gene Expression Assays (all from Applied Biosystems): Rn00582415_m1 (for Nrf2), Rn01751056_m1 (for MyhI), and Rn00567418_m1 (for MyoG). Actin beta (ACTB, 4352931) was used as endogenous control. Using the ΔΔC_t_ method, the expression of target (Nrf2, MyhI, and MyoG) mRNAs was normalized to ACTB mRNA and calculated as fold change relative to their expression in control cells.

### 4.9. Statistics

If not stated otherwise, results are presented as mean and standard error. One-way or two-way ANOVA followed by an appropriate post hoc test (Bonferroni’s or Dunnett’s test) was performed using GraphPad Prism (v6; GraphPad Software, Inc., La Jolla, CA, USA). Statistical significance is displayed as follows: ns, not significant (*p* > 0.05); * *p* ≤ 0.05; ** *p* ≤ 0.01; *** *p* ≤ 0.001; **** *p* ≤ 0.0001. All data generated or analyzed during this study are included in this published article.

## 5. Conclusions

By using in vitro models of an L6 rat skeletal muscle cell line and NPU cells that are able to differentiate or were already differentiated, we were able to evaluate the long-term in vitro toxicity of selected NPs. We showed that the sensitivity of L6 cells to NPs was much higher in comparison to NPU cells, which can be mainly attributed to the difference in internalization rate and specifically barrier function of the urothelial NPU cells.

In L6 cells, we have obtained a dose-dependent decrease in viability for acute and continuous exposure depending on the type of NPs. TiO_2_ NPs exhibited the most toxicity, with mild effects already at very low concentration after 10 days of continuous exposure. Altogether, the selected industrial SiO_2_ NPs did not induce cytotoxic effects, which can be explained mostly with large aggregation outside the cells under physiological conditions (aggregates sizes in the range of micrometers). The relatively stable NPs, the magnetic PAA NPs, had some moderate toxic effect, but only at extremely high concentrations despite very high intracellular loading. For continuous exposure, TiO_2_ and PAA NPs also induced the oxidative damage-associated response and hindered the differentiation of L6 cells, where again the effects were much more pronounced for TiO_2_ NPs. In this study, we demonstrate that more advanced in vitro models of cells that more closely resemble in vivo tissue and are capable of differentiation enable us to assess the long-term toxicity and testing of newly designed NPs. Thus, we propose an L6 rat skeletal muscle cell line and NPU cells as in vitro models that more closely resemble tissue properties and enable assessment of the long-term toxicity of relevant types of NPs that cross the barriers into blood or are secreted through the kidneys into urine.

## Figures and Tables

**Table 1:** Measured z-average size (by %Intensity), number-based hydrodynamic diameter (by %Number), polydispersity index (PDI), and zeta potential for polyacrylic acid (PAA), polyethylenimine (PEI), SiO_2_, and TiO_2_ nanoparticles (NPs) in distilled water and cell culture media used in the study.

NP Type	Dispersion Media	Z-average [nm]	Number-Based Hydrodynamic Diameter [nm]	PDI	Zeta Potential [mV]
PAA	distilled water	138 ± 48	64 ± 22	0.3± 0.1	−56.3 ± 6
	MEM alpha + 2% FCS	573 ± 333	62 ± 31	0.5 ± 0.0	−25 ± 2
	* UroM (+Ca^2+^ − S_FBS_)	1637 ± 461	239 ± 59	0.4 ± 0.0	−22.2 ± 1
PEI	distilled water	136 ± 25	104 ± 49	0.2 ± 0.1	54.4 ± 4
	* MEM alpha + 2% FCS	1352 ± 273	1075 ± 337	0.3 ± 0.1	−3 ± 1
	* UroM (+Ca^2+^ − S_FBS_)	1308 ± 39	1071 ± 327	0.3 ± 0.1	5 ± 2
SiO_2_ **	distilled water	515 ± 206	238 ± 70	.6 ± 0.2	2.4 ± 2
	MEM alpha + 2% FCS	145 ± 22	47 ± 2	0.4 ± 0.1	−9 ± 5
	UroM (+Ca^2+^ − S_FBS_)	2594 ± 303	88 ± 118	0.4 ± 0.1	NA
TiO_2_	distilled water	604 ± 300	201 ± 34	0.3 ± 0.1	28 ± 3
	* MEM alpha + 2% FCS	697 ± 244	240 ± 68	0.3 ± 0.1	−5.0 ± 5
	* UroM (+Ca^2+^ − S_FBS_)	1108 ± 559	293 ± 141	0.3 ± 0.0	−8.6 ± 0.7

* TiO_2_, PEI NPs in culture media, and PAA in UroM (+Ca^2+^ − S_FBS_) media defined in [40] formed aggregates in culture media and partially sedimented—the stable fraction was measured by dynamic light scattering (DLS). ** SiO_2_ NPs formed large aggregates >μm in diameter at pH 7 [4] that quickly sedimented, so the DLS measurements only represent a smaller fraction of these NPs.

## Supplementary Materials

The following are available online at https://www.mdpi.com/1422-0067/21/20/7545/s1, Figure S1: TEM micrographs of NPs dispersed in water, Figure S2: Number size distributions of NPs in distilled water, MEMa and UroM media.

## Abbreviations

NPs	nanoparticles
Co–ferrite	cobalt–ferrite
PAA	polyacrylic acid
PEI	polyethylenimine
PI	propidium iodide
ROS	reactive oxygen species
qPCR	quantitative polymerase chain reaction
Nrf2	nuclear factor (erythroid-derived 2)-like 2
MyoG	myogenin
MyhI	myosin heavy chain I
NPU	normal porcine urothelial
