# Supplementary material for: Proposing Urothelial and Muscle In Vitro Cell Models as a Novel Approach for Assessment of Long-Term Toxicity of Nanoparticles
Source: Int J Mol Sci. 2020 Oct 13;21(20):7545. doi: 10.3390/ijms21207545 (PMC7589566; doi:10.3390/ijms21207545)
Supplement: Supplementary file 1 [file ijms-21-07545-s001.pdf]

Supplementary material

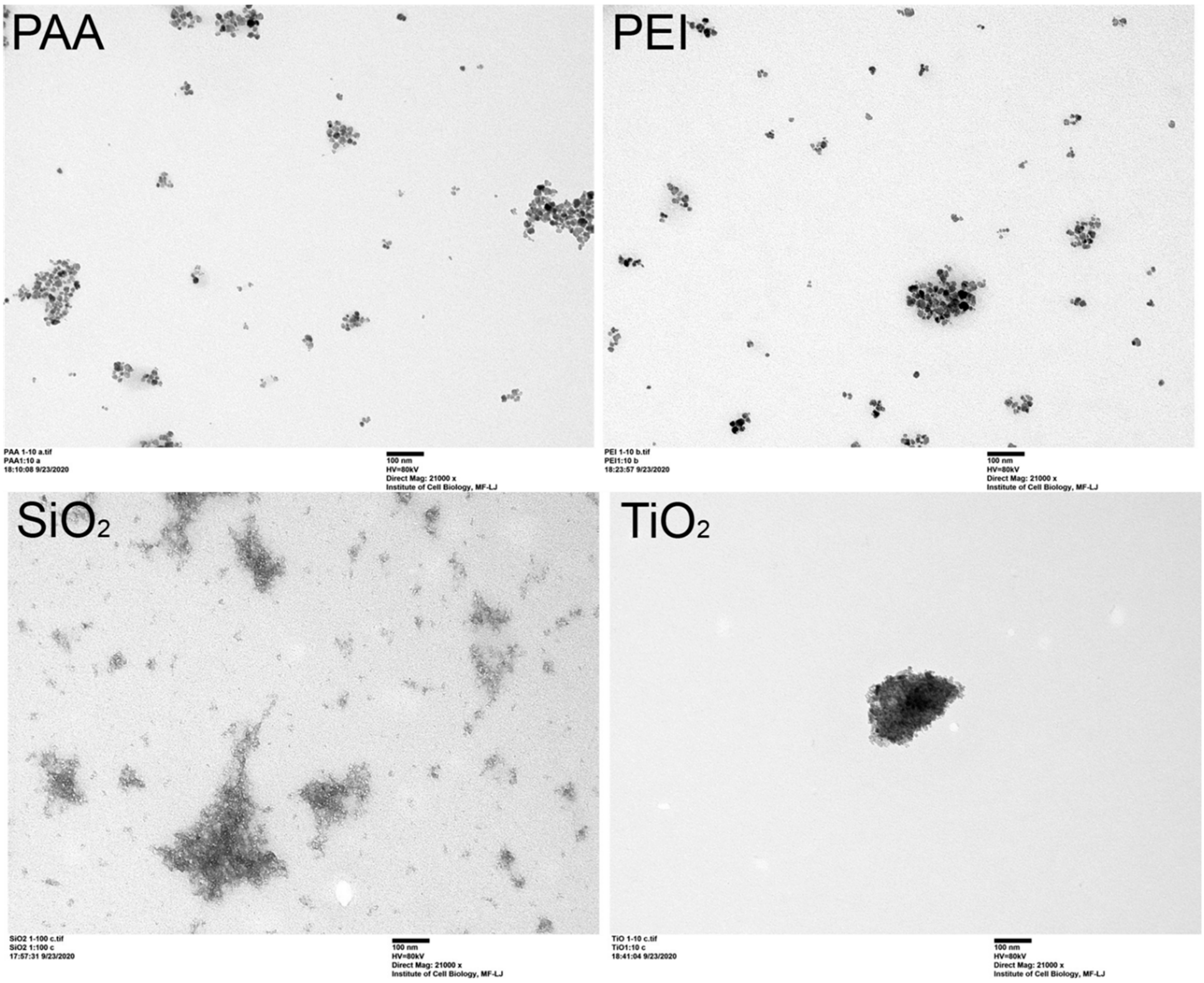

Figure 1. TEM micrographs of NPs dispersed in water. Scale bar: 100 nm.

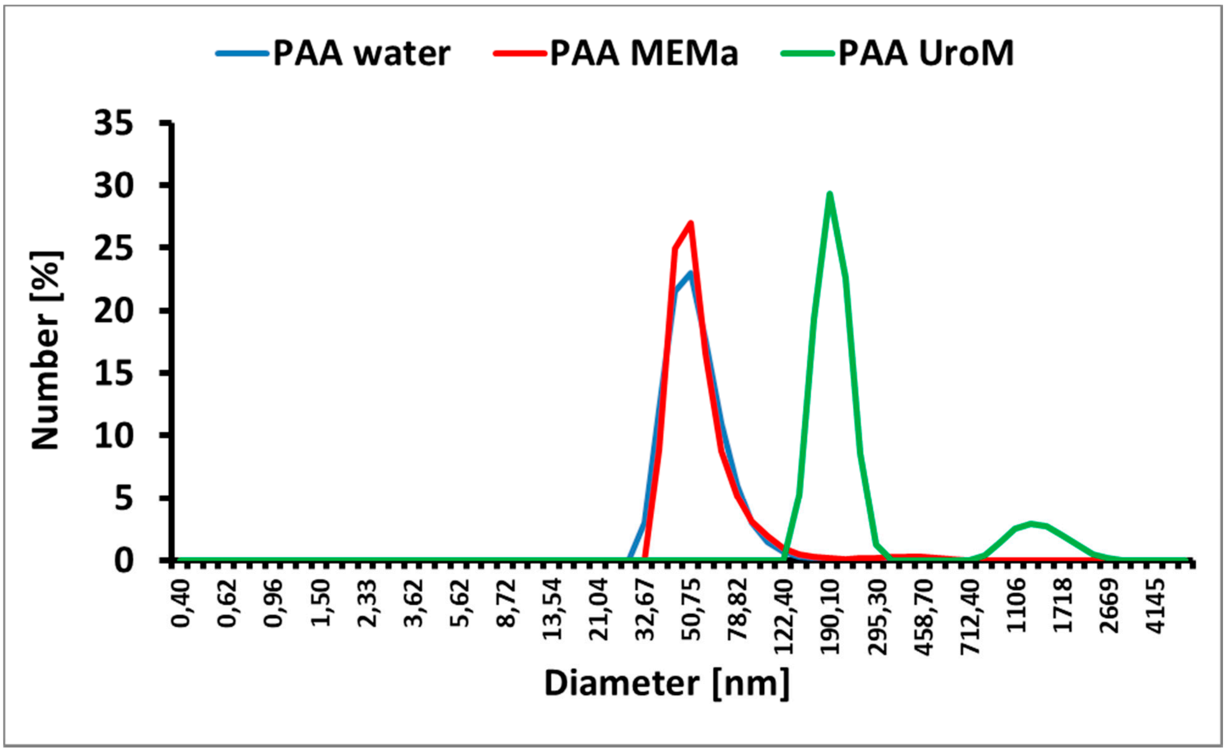
PAA water
PAA MEMa
PAA UroM
35
30
25
20
15
10
5
0
Number [%]
0,40
0,62
0,96
1,50
2,33
3,62
5,62
8,72
13,54
21,04
32,67
50,75
78,82
122,40
190,10
295,30
458,70
712,40
1106
1718
2669
4145
Diameter [nm]

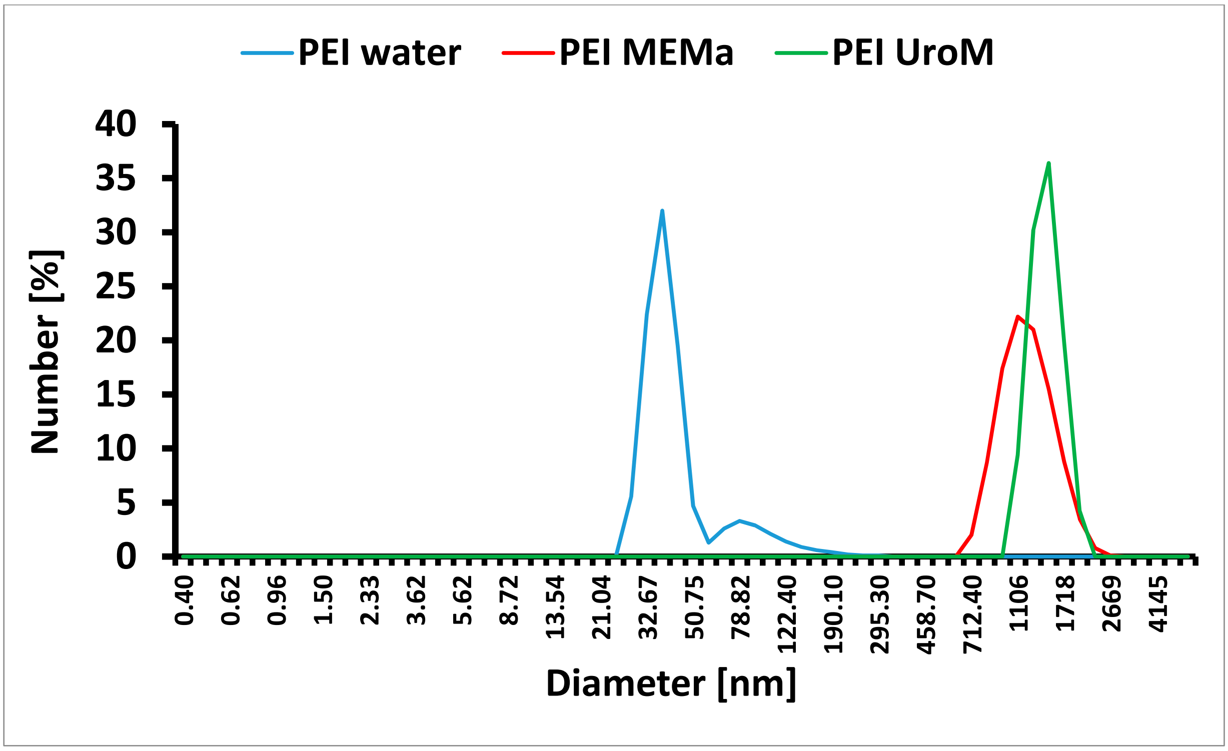
PEI water
PEI MEMa
PEI UroM
40
35
30
25
20
15
10
5
0
Number [%]
0.40
0.62
0.96
1.50
2.33
3.62
5.62
8.72
13.54
21.04
32.67
50.75
78.82
122.40
190.10
295.30
458.70
712.40
1106
1718
2669
4145
Diameter [nm]

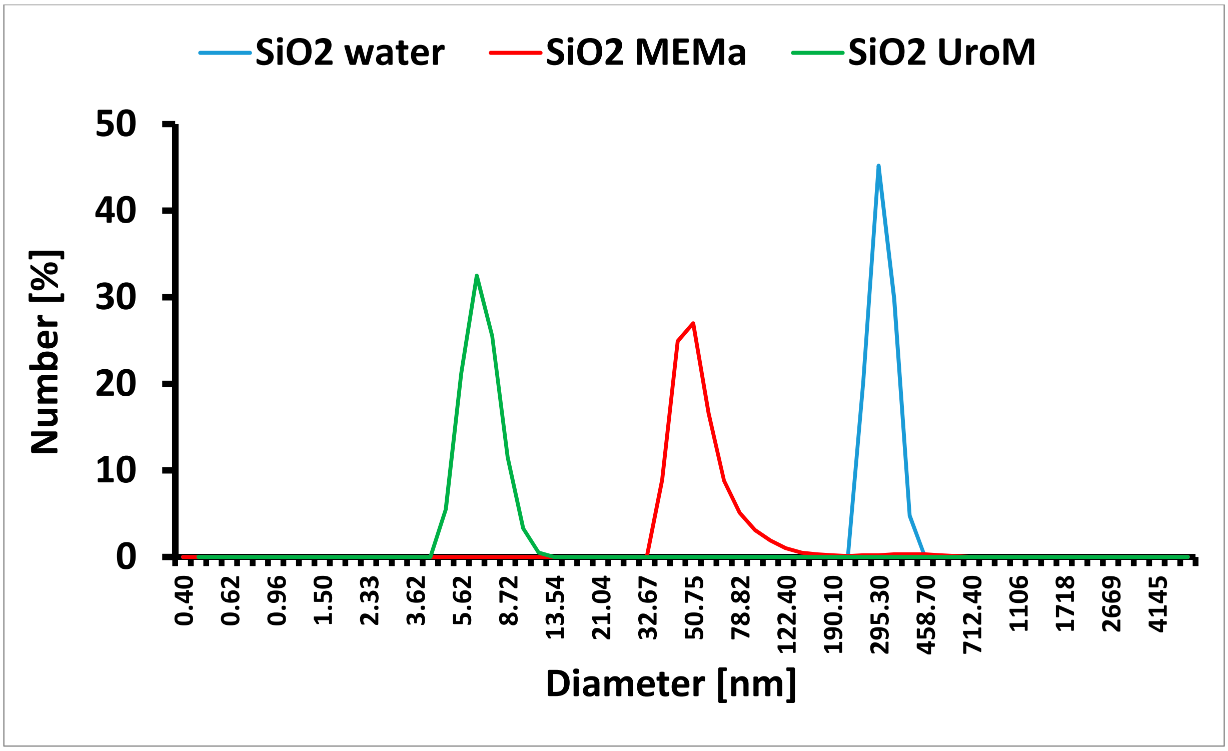

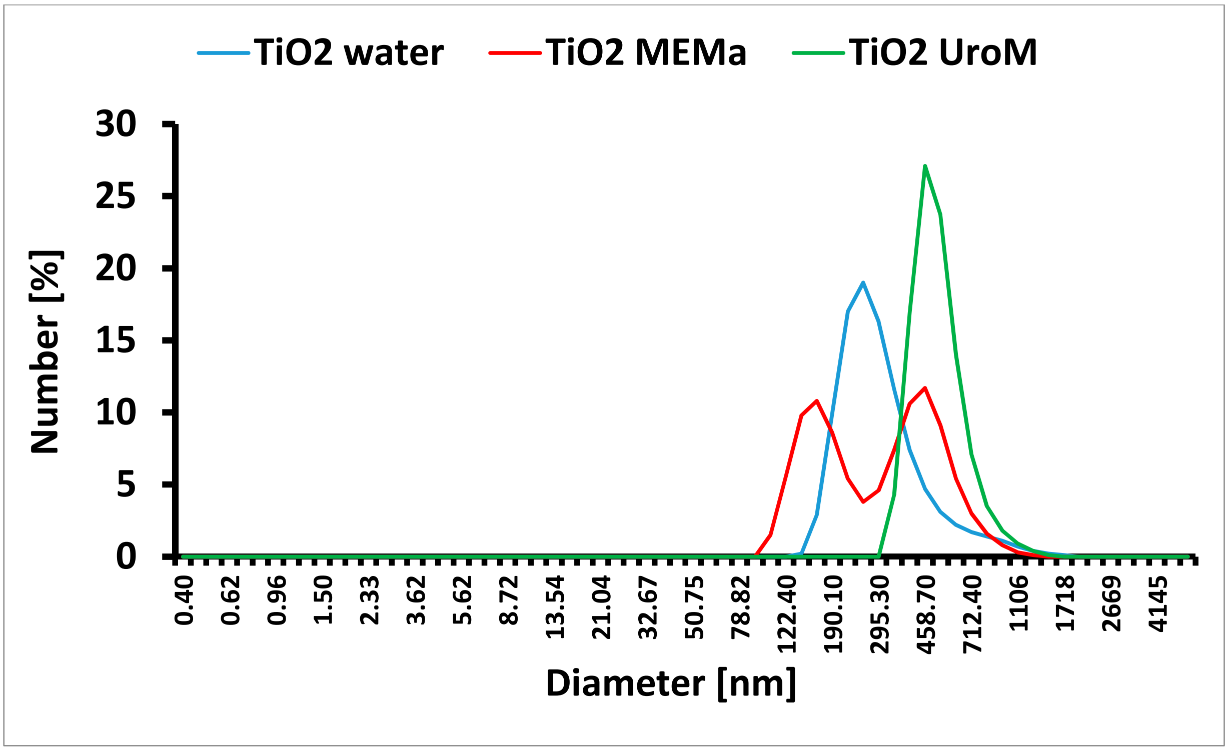

Figure S2: Number size distributions (one representative measurement) for PAA, PEI, SiO_2 and TiO_2 NPs in distilled water, MEM alpha media (MEMa) with 2%FCS and UroM +Ca-S_{FBS} media.
